# Application of AI in Cyberattack Detection: A Review

**DOI:** 10.3390/s26051518

**Published:** 2026-02-28

**Authors:** Yaw Jantuah Boateng, Nusrat Jahan Mim, Nasrin Akhter, Ranesh Naha, Aniket Mahanti, Alistair Barros

**Affiliations:** 1 Faculty of Physical and Computational Sciences, Department of Statistics and Actuarial Science, Kwame Nkrumah University of Science and Technology, Kumasi P.O. Box KS5013, Ghana; yjboateng@gmail.com; 2 Faculty of Science and Technology, American International University-Bangladesh (AIUB), Dhaka 1229, Bangladesh; mim786420@gmail.com; 3 School of Engineering, Swinburne University of Technology, Melbourne, VIC 3122, Australia; nakhter@swin.edu.au; 4 School of Information Systems, Queensland University of Technology, Brisbane, QLD 4000, Australia; alistair.barros@qut.edu.au; 5 School of Computer Science, The University of Auckland, Auckland 1010, New Zealand; a.mahanti@auckland.ac.nz

**Keywords:** artificial intelligence, cyberattack, intrusion detection systems, deep learning, federated learning, reinforcement learning, explainable AI, quantum computing

## Abstract

In today’s fast-changing digital environment, cyber-physical systems face escalating security challenges due to increasingly sophisticated cyberattacks. Artificial Intelligence (AI) has emerged as a powerful enabler of modern cyberattack detection, offering scalable, accurate, and adaptive solutions to counter dynamic threats. This paper provides a comprehensive review of recent advancements in AI-based cyberattack detection, focusing on Machine Learning (ML), Deep Learning (DL), Reinforcement Learning (RL), Federated Learning (FL), and emerging techniques such as generative AI, neuro-symbolic AI, swarm intelligence, lightweight AI, and quantum Computing. We evaluate the strengths and limitations of these approaches, highlighting their performance on benchmark datasets. The review discusses traditional signature-based Intrusion Detection Systems (IDS) and their limitations against novel attack patterns, contrasted with AI-driven anomaly-based and hybrid detection methods that improve detection rates for unknown and zero-day attacks. Key challenges, including computational costs, data quality, privacy concerns, and model interpretability, are analysed alongside the role of Explainable AI (XAI) in enhancing trust and transparency. The impact of computational resources, dataset representativeness, and evaluation metrics on AI model performance is also explored. Furthermore, we investigate the potential of lightweight AI for resource-constrained environments like IoT and edge devices, and quantum computing’s role in advancing detection efficiency and cryptographic security. The paper also draws attention to future research directions, particularly the development of up-to-date datasets, integration of hybrid quantum–classical models, and optimisation of asynchronous FL protocols to address evolving cybersecurity challenges. This study aims to inspire innovation in AI-driven cyberattack detection, fostering robust, interpretable, and efficient solutions for securing complex digital environments.

## 1. Introduction

In the fast-growing digital era, cyber-physical systems have become an essential element that drives innovation, thus enabling the integration of advanced technology in daily lives and business operations. The interconnectedness of the Internet of Things (IoT) along with the dependence of technology on vast data provides a re-imagination for industries, offering groundbreaking efficiency and multiple benefits [1]. However, this digital transformation has led to significant security challenges, with a meteoric rise in sophisticated attacks such as denial of service, information theft, phishing, and unauthorized access. Prevention is key to help avoid attacks on smart technologies for effective and continued system performance. Nonetheless, detection and mitigation measures are implemented to provide protection if prevention fails [2]. An Intrusion Detection System (IDS) is designed to detect and respond effectively to attempts at unauthorized access. This plays a critical role in cybersecurity by identifying suspicious attributes that may be a looming attack. Traditional IDS (signature-based) relies on predefined routines or signatures that match known malicious actions. Although very helpful, this system tends to struggle against emerging cyberattacks which continuously evolve with new attack patterns [1,3].

Artificial Intelligence (AI) advances have shown the potential to enhance IDS capabilities with the development and implementation of a variety of techniques, improving the ability to detect and respond to a wide range of cyber threats. In addition to enhancing signature-based detection, techniques such as anomaly-based detection by Machine Learning (ML) and hybrid detection are implemented to improve the accuracy and effectiveness of IDS [4,5]. Anomaly-based detection has the advantage of identifying new or unusual patterns, but it can be overly sensitive, often generating false positives even with slight deviations from normal behaviour. For further improvements, the development of a hybrid detection system where a signature-based method detects known threats and an anomaly-based method uncovers unfamiliar threats has been shown to reduce false positives and improve detection accuracy [3]. The use of AI for intrusion detection also raises several concerns; the creation of privacy concerns since AI requires a vast amount of data for training, the unavailability and difference of data across domains, the consistent updating and retraining of AI models to meet the evolving nature of cyberattacks, and the creation of ethical considerations from the use of AI in the context of critical operations [1]. Clearly, researchers continue to explore methodologies as the need to develop and advance more sophisticated IDS models to achieve operational efficiency in diverse and resource-limited domains grows [6].

**Figure 1:**
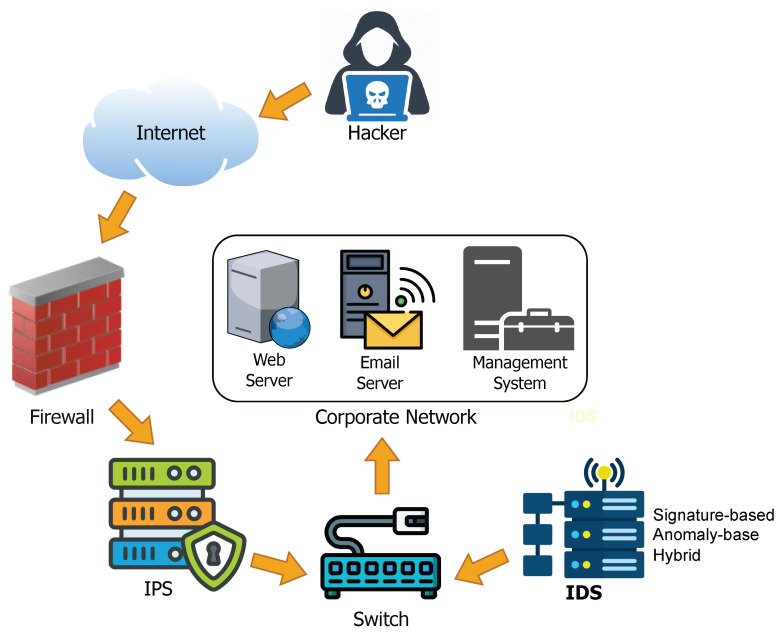
IDS in corporate network.

Figure 1 shows the role of IDS in the corporate network. The challenges posed by AI-driven detection inspire ongoing research into hybrid and intelligent cyberattack detection systems that provide effective, accurate, and scalable protection in complex digital environments. Hence, this paper aims to survey and evaluate recent and notable developments in different AI-based cyberattack detection. This paper presents a review of ML, Deep Learning (DL), Reinforcement Learning (RL) and Federated Learning (FL) algorithms applied to cyberattack detection, highlighting key challenges and outlining future research directions. The main goal of the study is to encourage innovation in cyberattack detection that can adapt to the evolving cyber-security landscape. The contributions of this paper are summarised as follows:

- Presentation of an overview of existing literature in cyberattack detection using AI techniques.
- Presentation of current developments in the use of ML/DL, FL and RL for detecting cyberattacks.
- Discussion on the impact of datasets and computational resources on AI techniques and systems, and the significance of evaluation metrics on the performance of these algorithms.
- Presentation on how lightweight AI and quantum computing can help improve cyberattack detection.
- Discussion on challenges based on current research and suggesting directions for the future.

## 2. Literature Review

AI has evolved into a crucial component in cybersecurity, enabling significant improvements in cyberattack detection. A primary application of AI is to address the limitations of traditional systems to enable a higher efficiency in threat detection [7]. Advancements in AI have emerged to address the ever-evolving landscape of threats and attacks. Initially, cyberattacks classification relied on the development of ML techniques, which leverage data-driven models to improve detection accuracy. DL methods were then adopted to further improve the algorithm performance by transforming raw data into appropriate formats as traditional ML techniques are constrained in their ability to process raw data effectively and therefore depend heavily on well-designed feature extraction [8,9]. When data privacy and security are critical, FL is a distributed Machine Learning paradigm that allows multiple participants to collaboratively train models on large-scale data while keeping raw data local, avoiding direct sharing with a central server or other entities [10]. Currently, a promising direction for attack detection has increasingly drawn on RL techniques, a technique that aims to learn an optimal policy strategy during the model training process. RL has shown significant potential to enhance reasoning abilities [11,12]. Nonetheless, the development of these learning algorithms also presents critical challenges, including scalability, generalizability, robustness of the model, security, and privacy [13]. These factors motivate the need for continuous learning to increase the effectiveness of AI in cybersecurity. To further enhance the potential of AI systems, researchers continue to explore their application in detecting cyberattacks. This study presents several AI-based attack detection research works. Comparison to existing literature in different aspects of AI, i.e., ML, DL, RL and FL are discussed.

Rahman et al. [4] present a comprehensive survey of recent IDS research, offering a broad performance-driven overview of prevailing techniques and models. Their analysis shows that most studies concentrate on traditional Machine Learning approaches, ensemble methods, Deep Learning models, neural network architectures, and hybrid frameworks. Widely adopted classifiers such as Random Forest, Gradient Boosting Machine, Naive Bayes, AdaBoost, and Logistic Regression are frequently selected due to their robustness and consistent classification performance. In parallel, Artificial Neural Networks, including specialised architectures such as Convolutional Neural Networks and Deep Neural Networks, have proven effective in capturing complex and high-dimensional data patterns. The survey also highlights the use of algorithms such as k-nearest neighbours, Decision Trees, and Support Vector Machines, alongside advanced ensemble strategies like Extreme Gradient Boosting and stacking, which contribute to improved feature selection and classification accuracy. In addition, bio-inspired optimisation techniques, including Particle Swarm Optimisation, Genetic Algorithms, and hybrid variants such as GA-GWO, have been explored to enhance model efficiency. The integration of data balancing techniques such as SMOTE, combined with feature selection and hybrid detection models, reflects the growing sophistication of IDS research, particularly in Internet of Things environments. Emerging approaches based on Variational Autoencoders and Deep Convolutional Generative Adversarial Networks further signal a shift towards more complex and expressive detection models. Despite these advances, the survey notes that several challenges remain, and the effectiveness of certain Machine Learning techniques for attack detection requires further investigation [4].

Building on this foundation, the literature indicates that Federated Learning is rapidly gaining attention due to its privacy-preserving and collaborative nature. Vanilla Federated Averaging remains the most widely adopted aggregation strategy, with expectations that ongoing improvements in FL will significantly benefit IDS by leveraging distributed and cooperative learning. Existing federated anomaly detection frameworks are viewed as a strong basis for developing more advanced FL-IDS solutions. However, current state-of-the-art FL-IDS approaches continue to rely predominantly on anomaly-based detection, despite the availability of alternative IDS architectures. Key challenges identified include time constraints, inefficient task handling, and the lack of standardised datasets, all of which hinder robust FL model development. The survey further emphasises the need for reliable and standardised evaluation metrics to enable fair and meaningful comparisons across FL-IDS studies. A notable trend in recent work is the growing use of Bidirectional Long Short-Term Memory models for streaming data scenarios, underscoring the expanding scope of FL-based IDS research. Nevertheless, real-time data evolution remains in its early stages, and detecting novel threats continues to pose significant difficulties. To address these issues, the survey advocates for weight-based aggregation mechanisms to accelerate model convergence and stresses that next-generation FL-IDS must incorporate self-security mechanisms to mitigate risks such as model poisoning and adversarial manipulation [14].

Other related reviews conducted due to the growing importance and application of AI for cyberattack detection, report on similar algorithms used in research, including supervised and unsupervised ML and DL [15,16,17]. While some reviews present a detailed taxonomy on IDS types [15], others present a detailed submission of learning methods [16]. Dataset limitations, lack of standard evaluation metrics, computational efficiency seem to be a common challenge found in almost all reviews. Another review offers a comprehensive overview on the application of RL and Deep RL in enhancing IDS across communication networks and cloud environments. Upon highlighting the limitations of traditional IDS, the review emphasizes how RL/DRL offers intelligent, adaptive and real-time responses to evolving cyber threats. After reviewing attacks and datasets, and performance metrics, the survey presents an extensive taxonomy of RL/DRL techniques, analysing over various relevant works and covering recent developments up to 2024. Additionally, research gaps such as scalability, computational efficiency, and adversarial robustness as in most reviews were identified. Federated and adversarial RL, lightweight models, and improved interpretability were among the recommendations for future directions [13]. Overall, these surveys and reviews serves as a state-of-the-art reference for researchers and practitioners, valuable for advancing cyberattack detection in the increasingly complex digital space.

As noted in Table 1, most related reviews focus on a specific AI field, highlights challenges and suggests future directions. Another notable shortcoming in existing reviews is the discussion of evaluation metrics. While they are fully reviewed in some of the literature, some studies partially discuss it and most studies do not. This study then seeks to assess the current developments of ML and DL, FL, and RL in cyberattack detection to help improve existing literature in the domain. Evaluation metrics of the models will also be discussed to address challenges and suggest improvements. In addition, this study discusses recent developments in lightweight AI and how quantum computing can help advance cyberattack detection.

**Table 1:** Summary of existing related works.

Reference	Year	AI Focus Field	Discussions			
			**Datasets**	**Evaluation Metrics**	**Challenges**	**Future Directions**
Kaloudi and Li [18]	2020	Unspecified	x	x	√	√
Sharma et al. [19]	2024	ML, DL	x	x	x	x
Abdullai et al. [20]	2022	ML, DL	Partially	x	√	√
Siam et al. [21]	2025	ML, DL	x	x	√	√
Sowmya and Mary Anita [16]	2023	ML, DL	Partially	Partially	√	√
Salem et al. [22]	2024	ML, DL	√	x	√	√
Ahmetoglu and Das [17]	2022	ML, DL	√	√	√	√
Makris et al. [1]	2025	FL	√	Partially	√	√
Al-Shurbaji [23]	2025	DL	Partially	Partially	√	√
Naghib et al. [15]	2025	ML, DL	√	√	√	√
Belengeur et al. [14]	2025	FL	√	√	Partially	√
Rahman et al. [4]	2025	ML, DL	√	√	√	√
Kheddar et al. [13]	2024	RL	√	x	√	√
Yang et al. [24]	2022	ML, DL	√	√	x	x
Fedorchenko et al. [25]	2022	FL	x	Partially	x	x
Yang et al. [26]	2024	DRL	√	x	x	√
Our Work	2025	ML, DL, FL, RL	√	√	√	√

## 3. Current Development in Cyberattack Detection

**Figure 2:**
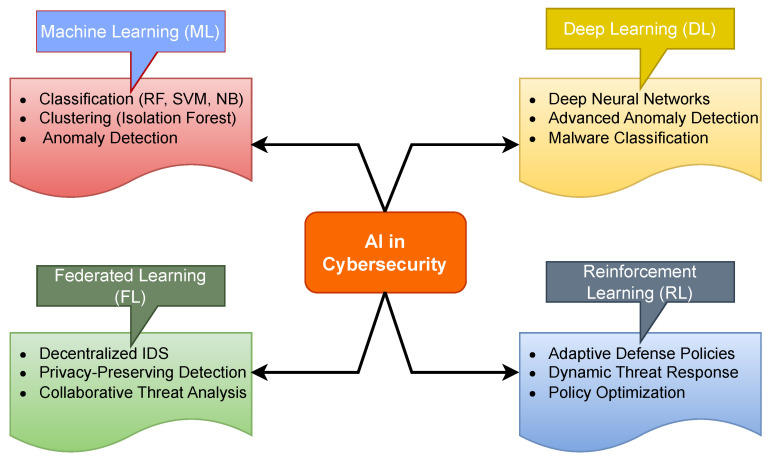
Application of ML, DL, FL and RL in cybersecurity.

This section discusses the application of AI techniques in cybersecurity. Brief descriptions on the overview of ML, DL, FL and RL techniques and algorithms are given, and their application in cyberattack detection is reviewed. Figure 2 presents an overview of major Artificial Intelligence paradigms applied in cybersecurity. The diagram illustrates how AI-driven security solutions are structured around four principal approaches: ML, DL, FL, and RL. ML techniques support tasks such as classification, clustering, and anomaly detection using algorithms including Random Forest (RF), Support Vector Machine (SVM), and Naïve Bayes (NB). DL enables advanced anomaly detection and malware classification through deep neural architectures. FL facilitates decentralised and privacy-preserving intrusion detection across distributed environments, while RL contributes adaptive defence strategies and dynamic policy optimisation. Collectively, these approaches demonstrate the diverse methodological landscape underpinning modern AI-based cybersecurity systems.

The paper then discusses recent developments of these techniques and highlights their performances in studies. This paper also considers the evolution of datasets in cybersecurity, highlighting the challenges encountered from the use of benchmark datasets and the significance of generating new high-quality datasets to meet real-world expectations. The importance of performance evaluation of AI models is discussed. This work highlights both established and proposed metrics, underscoring the significance of standardised evaluation in performance assessment. Finally, the paper compares the computational resources used in various studies and briefly discusses the impact of these resources on the performance on AI implementations.

### 3.1. Comparative Overview of AI Paradigms for Cyberattack Detection

Before discussing each paradigm in detail, Table 2 provides a structured comparison of the considered AI techniques in terms of their applicability to different attack types, computational requirements, training complexity, data dependency, interpretability, and deployment suitability. This comparative perspective clarifies the operational distinctions between traditional ML/DL approaches, adaptive RL-based methods, privacy-preserving FL systems, emerging generative and neuro-symbolic frameworks, and quantum-enhanced models.

### 3.2. ML/DL Models for IDS

ML has gained wide interest in cybersecurity, particularly to analyse and classify bad actors from huge datasets. Various ML algorithms and approaches exist, broadly being categorised into supervised and unsupervised learning. Both approaches can be applied to analyse malware in near real-time to help eliminate the flaws of traditional models. Supervised learning involves classification, where inputs are matched to specific outputs, or regression, which predicts continuous output from inputs. On the other hand, unsupervised learning is used for exploratory analysis and dimension reduction, primarily through clustering [27]. Recently, DL algorithms have successfully been experimented on supervised and unsupervised data for cyberattack detection, providing expected solutions with good performance [28]. The abilities of ML and DL are continuously being explored in various research.

A lightweight Intrusion Detection System combined with a feature selection approach based on simple statistical measures was shown to improve accuracy and overall performance, particularly for classifiers that initially performed poorly. Comparative evaluations of Machine Learning techniques for IDS indicated that ensemble methods such as Random Forest and AdaBoost consistently outperformed more traditional algorithms. The experimental analysis included both advanced classifiers, namely Random Forest and AdaBoost, as well as simpler models such as Logistic Regression, Support Vector Machines, Naive Bayes, and Decision Trees. Results demonstrated that the simpler classifiers generally exhibited lower accuracy and reduced efficiency in distinguishing malicious activity. The application of the Transfer Learning approach on future ML works is recommended as well as using it domain-specific pre-trained models. Furthermore, the study suggested that the use of DNN may reduce the intrusion detection time [29]. In attempts to improve ML in cyberattack detection, hybrid models are also being introduced in some studies. Attri et al. [3] introduced a hybrid ML system in order to reduce false positives and improve the detection accuracy in the detection of network intrusions. Isolation Forest, a proven effective malicious behaviour identifier without prior knowledge of attack signatures, was added to the usual signature-based ML method. This method, as demonstrated by several studies, is considered highly efficient in detecting anomalies in high-dimensional datasets, making it ideal for network intrusion detection. Even though the hybrid system is effectively executed to improve cybersecurity, the article did not provide detailed performance metrics, making it difficult to quantify improvements. The exploration of unsupervised or semi-supervised techniques was recommended to further enhance the accuracy and adaptability of the system [3]. In other cases, Hybrid ML models in different forms have been proven to be a promising solution for the detection of complex and evolving cyber threats, as it outperforms traditional model techniques. Addressing the ever-changing cybersecurity landscape and developing its intrusion detection capabilities can be the future scope of the hybrid model, although it is believed to be years ahead of traditional models [2,5].

The research reviewed in Table 3, explores various ML and DL techniques to enhance the performance of cyberattack detection, focusing on efficiency, improved accuracy, reduced false alarms and adaptability. Several common methodologies emerge across studies. A plethora of algorithms were employed, including traditional ML models like SVM, KNN, RF, DT, NBC, LR, and Linear/Quadratic Discriminant Analysis. Advanced techniques like GBM, Light GBM, XGBoost, ANN, LSTM, Recurrent Neural Network (RNN) and Graph Neural Networks (GNN) are also recognised. Evaluation of these models were mostly implemented on benchmark datasets, with emphasis placed on crucial preprocessing steps such as handling missing values and duplicates, encoding categorical features and normalisation and addressing class imbalance, mostly using SMOTE [5,30]. Several studies project the significance of reducing dimensionality and selecting relevant features to improve model efficiency and accuracy. Techniques such as correlation analysis, visualization, Recursive Feature Elimination (RFE) combined with classifiers, bio-inspired algorithms, and fuzzy clustering were employed in studies. The performance and robustness of models were shown with combined multiple classifiers through techniques like stacking or using inherent ensemble models [5,30,31]. Metaheuristic algorithms such as Particle Swarm Optimization (PSO), JAYA, Salp Swarm Algorithm (SSA), GWO, and Quantum Binary Bat Algorithm (QBBA) are used to optimize model hyperparameters or feature selection processes [2,30,32].

**Table 3:** Summary of ML/DL models used in research.

Ref.	Proposed Methodology	ML/DL Models	Performance
Alotaibi et al. [2]	A network IDS model that uses a mix of bio-inspired metaheuristic algorithms to identify generic attacks	Grey Wolf Optimisation Algorithm, Quantum Binary Bat Algorithm, Naive Bayes, KNN, RF	The model reduced the number of features used for classification while keeping high accuracy, sensitivity, and F-score across the board
Rajathi and Rukmani [5]	A Hybrid Learning Model (HLM) that combines parametric and non-parametric classifiers	KNN, DT, RF, Gradient Boosting, SVC-RBF, LR, Naïve Bayes, Linear and Quadratic Discriminant Analysis, Linear SVM	The HLM achieves a better accuracy rate compared to existing models and shows a significant reduction in false alarm rate across various datasets
Saravanan et al. [33]	A Deep Learning algorithm to detect possible attacks on MANETs nodes	GNN	The simulation shows better resilience to network attacks than other methods
Ahmed et al. [30]	To improve network security by combining methods for intrusion detection from ML and DL	SVM, KNN, RF, DT, LSTM, ANN	All tested models can tell the difference between normal and intrusive behaviours and are very effective against complex intrusions
Dash et al. [32]	An optimized Long Short-Term Memory (LSTM) model for identifying anomalies in network traffic	Particle Swarm Optimisation, JAYA, and Salp Swarm Algorithm	The simulation results show that SSA-LSTM IDS outperforms all models considered in this study across three datasets
Almania et al. [31]	A new adaptive IDS that reduces the impact of outdated models and imbalanced datasets	KNN, Fuzzy c-means clustering, weight mapping, DT, RF	The proposed model achieves high accuracy with a low false alarm rate, outperforming traditional IDS models
Logeswari et al. [34]	A new Hybrid Feature Selection IDS for Software Defined Networking	LGBM	The proposed systems deliver excellent results compared to existing methods in terms of performance metrics
Attou et al. [35]	A cloud-based anomaly detection model	RF	The approach shows good performance metrics across datasets when compared to recent related works

Comparatively, studies that used ensemble or hybrid approaches consistently demonstrated high performance, achieving over 99% accuracy across different datasets in one instance [5] and reaching 97.7% accuracy in another study [31]. Stacking is shown to leverage the strengths of diverse base and meta-learners. DL models like LSTM, ANN and GNN show good performance at capturing complex and temporal patterns. SSA-optimised LSTM achieved accuracy as high as 99.8% [32]. GNNs showed superior performance in MANET simulations based on Packet Delivery Ratio (PDR) and delay metrics [33]. Although DL models are very powerful, they often require careful hyperparameter tuning, which optimisation algorithms like SSA can effectively provide [32]. Traditional ML models like RF, SVM, and KNN also show strong results, often with better interpretability [30,35]. In particular, RF performed well across multiple studies, sometimes achieving the highest accuracy comparatively between 98.3% [35] and 99.5% [2]. Reviewed studies consistently show that selecting optimal features subsets improve model accuracy and reduces computational overhead. A hybrid model successfully reduced features to 12 while achieving 98.5% accuracy with RF [2]. Another hybrid feature selection method used in a study led to high performance as compared to single methods [34]. Visualisation-based selection also proved effective, reducing features to 2 while achieving 98.3% accuracy with RF [35].

In summary, the potential of ML and DL techniques to overcome the limitations of traditional IDS has been collectively highlighted in the studies. These advanced methods can enable the handling of large-scale data, learn and adapt to evolving and complex attack patterns more effectively than traditional systems. Many proposed models achieve accuracy rates exceeding 95%, and often reaching over 99% on standard datasets. DL models, especially LSTM and GNN, are proficient at understanding patterns over time and structural relationships in network data. Feature selection and optimised algorithms such as LightGBM and optimised LSTM help to improve computational efficiency. Adaptability to dynamic traffic and imbalanced data is made easier with techniques like regulated retraining and ensemble methods.

### 3.3. Federated Learning IDS

Federated Learning, a technique that enables multiple entities and environments to learn a common AI model together while keeping all the training data localised, has gained special attention in technology for its reduction in the cost of communication and privacy preserving abilities [1,14]. There are unanimous considerations from existing literature that FL presents commendable advantages with respect to scalability, privacy and fault tolerance; perfecting the aim of IDS and making them more efficient and robust. The most important aspects of FL architectures noted are included in a multidimensional classification with learning models, privacy mechanisms, communication architectures, scale of federation, data partitioning and motivation of federation. FL systems are capable of managing a large variety of ML models with no boundaries, from simple models to more complex approaches. Linear models, DTs and neural networks are some of the most common categories of learning models usually employed for FL. The parameters of the learned model is not allowed to exchange sensitive data to help prevent inversion or inference attacks. Different privacy mechanisms such as cryptographic methods and differential privacy, are presented in order to achieve this. Centralised and decentralised communication architectures have emerged based on the mode or pattern in which information is exchanged. The scale of federation is determined by the number of parties and usage of computing resources during the learning process; namely, cross-silo (limited participants with high computational resources) and cross-device (more participants with limited resources). Existing data partitioning schemes are horizontal, vertical and hybrid, and this is dependent on the manner in which participants split data. Individual parties may be motivated to enrol based on incentives or regulations [14].

From Table 4, notable advantages of FL as highlighted in reviewed studies include preserved privacy, reduced communication costs, collaborative learning and allowance for potential adaptation of the global model to specific local data characteristics. A range of methodologies with the FL-IDS framework were employed: Various ML/DL models such as CNN, RNN, LSTM, Gated Recurrent Units (GRU), AE, DNN and advanced Multimodal Large Language Models (LLMs) were used for local participant training. Algorithms like PSO, JAYA, SSA and Randomised Search were employed for hyperparameter optimisation in studies while feature selection techniques included the use of RFE, Correlation-based Feature Selection (CFS), Chimp Optimisation Algorithm (COA) to improve model performance and efficiency. FedAvg algorithm is noted to be the most used fusion technique in studies. Logit adjustment technique was used to specifically optimise personalised FL models for heterogeneous data. Even though FL provides a privacy advantage, some studies incorporated differential privacy by adding noise to updates and permissioned blockchains for creating secure, auditable trails of model updates. Other specific defence mechanisms were also developed to detect and mitigate poisoning attacks. In reviewed studies, FL-based IDS generally achieved high detection accuracy, often comparable to centralised approaches albeit being slightly lower than them sometimes, exhibiting FL’s viability while preserving privacy. Reported accuracies differentiated between 92% and 99% based on the dataset, model and FL setup [36,37,38]. Unsupervised models like AE performed well in FL systems for anomaly detection. CNN was frequently used in studies and proven to be effective. Similarly, Multimodal LLM integrated with FL presented high accuracy on complex, heterogeneous datasets. Personalised FL models also showed improved performance, especially on non-Identically and Independently Distributed (non-IID) data. The performance of feature selection techniques increased significantly by reducing data dimensionality. Efforts to handle challenges associated with FL including data heterogeneity and poisoning attacks were noted. Non-IID data generally degrades performance compared to IID scenarios. However, personalisation techniques like logit adjustment loss and robust aggregation algorithms like Federated Proximal (FedProx) helped mitigate this issue. The effectiveness, however, varied between studies. Although Federated Learning may experience reduced performance under highly non-IID conditions, it remains effective when participating clients share partially overlapping feature spaces and observe broadly similar traffic patterns, even if class proportions differ. Such moderate heterogeneity can typically be managed through appropriate aggregation or regularisation strategies. Significant degradation generally occurs only when data distributions are substantially divergent, such as when clients encounter entirely different attack types. Therefore, FL is most advantageous in distributed settings where data similarity exists alongside privacy or regulatory constraints that limit centralised training.

Studies confirmed FL’s vulnerability to data and model poisoning attacks launched by malicious clients. Standard robust aggregators like median, trimmed mean, and Krum provided some defence, but they often struggled, especially with non-IID data. Specific defence mechanisms, like the two-phase client similarity alignment in personal FL models (pFL-IDS), proved more effective at detecting and excluding malicious updates. Using a permissioned blockchain, specifically MultiChain to record FL model updates was shown to be feasible for creating an auditable and tamper-resistant system with a manageable performance overhead, estimated at 5% to 15% [39].

In practical cybersecurity settings, direct training of Reinforcement Learning agents on live attack streams is rarely feasible due to operational risk and ethical considerations. Consequently, most RL-based IDS studies rely on controlled simulation environments, traffic replay systems, or digital twin testbeds that approximate network dynamics. While this approach differs from fully interactive real-world Reinforcement Learning, it enables policy optimisation in a safe and reproducible manner. True online adaptation is typically introduced only after extensive offline training and validation. The reviewed studies collectively highlight FL as a powerful paradigm for developing IDS in distributed environments, effectively balancing privacy requirements with the need for collaborative model training. The ability to train on diverse, localised datasets without central pooling is a significant advantage, particularly for IoT where data is generated at the edge and may be sensitive or voluminous.

While AI-driven Intrusion Detection Systems aim to identify malicious behaviour, the models themselves may become targets of adversarial manipulation. Attackers can attempt evasion by crafting adversarial network traffic designed to mislead classifiers, conduct data poisoning to corrupt training datasets, or perform model extraction to replicate decision boundaries. These threats highlight the need to consider IDS robustness as a core design requirement rather than an afterthought. Robustness verification typically involves stress-testing models under adversarial perturbations and evaluating their stability against manipulated inputs. Defence mechanisms include adversarial training, input sanitisation, anomaly-based filtering of training data, regularisation techniques to reduce model sensitivity, and secure aggregation in distributed learning environments. Incorporating these safeguards enhances the resilience of IDS frameworks and mitigates risks associated with adversarial exploitation.

**Table 4:** Summary of FL techniques in other research.

Ref.	Proposed Methodology	Fusion Technique	Performance	Datasets
Olanrewaju-George and Pranggono [40]	Use of unsupervised and supervised DL models trained via FL to develop IDS for IoT devices	FedAvgM	Effectively improved the performance and privacy of IDS for IoT devices	N-BaIoT
Wang and Yang [41]	A new distributed security threat detection system that combines Federated Learning with multimodal large language models	Weighted Summation	Maintains efficient processing capabilities in distributed environments and achieves higher detection accuracy while reducing both FP and FN rates	Not specified
Rashid et al. [36]	A FL method for detecting unwanted intrusions to ensure the protection of IoT networks	FedAvg	Achieves competitive results in intrusion detection, demonstrating its applicability and usefulness, and has significant effects for using FL in IoT networks.	Edge-IIoTset
Abdeldjalil and Mustapha [37]	A unified learning-based Intrusion Detection System using a neural network algorithm	FedAvg, FedProx, FedAdagrad, FedAdam	The algorithm showed good results on the dataset using both central and decentralized approaches while ensuring data privacy and model security in the decentralized approach	UNSW-NB15
Karunmurthy et al. [38]	A FL-based Intrusion Detection System that trains Deep Learning classifiers in IoT networks to identify different attacks	FedAvg	The model provides the highest intrusion detection accuracy compared to traditional ML algorithms	MQTT dataset
Presuveneers et al. [39]	A solution where contributing parties in Federated Learning can be held accountable and have their model updates reviewed	Custom technique	Illustrates that the added complexity from blockchain technology has a limited impact on the performance of FL while providing full transparency over the distributed training process of the neural network. Additionally, the blockchain-based FL solution can be generalized and applied to more complex neural network architectures and other use cases.	CICIDS2017
Mohammed et al. [42]	A complete solution to tackle the complex issue of protecting IoT environments by combining FL and IDS	FedProx, FedAvg	The approach effectively safeguards privacy and minimizes false alarms while ensuring effective detection of network intrusions	USTCTFC2016, CICIDS2017, and CSE-CIC-IDS2018
Thein et al. [43]	A personalized FL-based IDS approach to manage imbalanced data distributions and counter poisoning attacks	pFL, FedAvg	The approach successfully detects poisoning attacks without sacrificing performance regardless of the data distribution from the client	N-BaIoT

### 3.4. Reinforcement Learning for IDS

Reinforcement Learning is a learning paradigm in which an agent discovers optimal actions through trial-and-error interactions with its environment, guided by feedback in the form of rewards or penalties [44,45]. Unlike other Machine Learning approaches, Reinforcement Learning explicitly focuses on sequential decision-making, continuously refining its policy by learning from the ongoing interaction between the agent and the environment in order to maximise long-term returns [46]. This gives RL significant advantages over ML techniques for threat detection. RL’s effectiveness has been proven constantly through its ability to solve complex problems and dynamically adapt to the ever-changing environments, making it a powerful and suitable approach for feature selection and threat detection, which is an important role for detecting and mitigating threats in these complex systems. Agents develop the skill to identify the most relevant features through repeated trial and error, thereby increasing classification efficiency and limiting computational overhead. RL is also capable to continuously scale large and complex networks and data distributions, making it a suitable tool for securing systems and a core advantage for dynamic feature selection. However, it may require a vast amount of data to learn effectively compared to other techniques. Some of the most common RL algorithms used in solving challenges include Q-learning, State–Action–Reward–State–Action (SARSA), Deep Deterministic Policy Gradient (DDPG) and Advanced Deep Reinforcement Learning (ADRL). Notably, Q-learning has been a popular choice for various research projects [45,47]. A valuable tool for network security is the robustness and generalizability of an RL-based approach. This is an important factor to consider when evaluating its performance, and has the potential to be more effective at detecting and mitigating threats in a wide range of different circumstances and environments. An evaluation of the robustness and generalizability of an RL-based approach will be to analyse its performance on a wide range of different tasks and environments. Weaknesses and limitations of the approach and areas where it is ineffective can be identified this way. Furthermore, evaluating the performance of an RL-based approach on similar but not identical tasks can be an alternative way to assess its robustness and generalizability. This can help to determine whether the approach is able to generalise its knowledge to new situations and to identify any areas where it may be less effective at adapting to new environments [45].

Several studies in Table 5, explored RL and DRL as promising approaches for intrusion detection with goals to overcome the limitations of traditional ML methods due to its significant advantage over static models. RL agents have the ability to learn and adapt to dynamic network environments and evolving threats. RL also allows for more autonomous detection and response, potentially reducing the need for manual rule updates or frequent retraining. Again, RL methods show potential in detecting unknown or zero-day attacks by learning anomalous patterns rather than solely depending on known patterns. Algorithms explored across studies included Deep Q-Network (DQN), Double DQN (DDQN), SARSA, Policy Gradient (PG) and Actor-Critic, specifically Advantage Actor-Critic (A2C). Some studies modified the standard RL framework, for instance by using labelled datasets instead of live interaction, simulating the environment, and defining rewards based on classification correctness or errors [11,44,48].

**Table 5:** Summary of RL-based research works.

Work	Proposed Methodology	RL Algorithm	Performance
Saeed et al. [45]	Detected threats in dynamic edge network environments using a real-time threat detection system based on intelligent RL	DQN, SARSA, Q-Learning	The method surpasses traditional techniques in the detection of threats in edge network environments that are changing
Shyaa et al. [47]	A novel Incremental Feature Drift-Aware Genetic Programming Combiner to handle feature drift and maintain accuracy	VE-DQN	The framework guarantees that feature drifts in real-time are managed with consistency and reliability while offering top-notch classification performance
Kim et al. [44]	Implementing RL methodology, Actor-Critic, and NLP for the extraction of keywords that appear on each anomaly system call log and proposing a rule generation framework to stop future intrusion detection by using the extracted words	Advanced Actor-Critic	The method has an average accuracy rate that is relatively high when dealing with different attack logs
Roy and Kalita [46]	Integrating an improved Deep RL model into an intelligent semi-supervised IDS using various algorithms for precise classification of network attacks to extract high-level representations and learn complex patterns from data	DAE, IFSA	Framework achieves better accuracy across datasets than current detection systems
Santos et al. [11]	A new model of intrusion detection based on a RL approach that seeks to support long periods without model updates	Q-learning	The technique without continual model updates allots similar accuracy rates to conventional detection schemes. It lowers false positives and negatives while increasing accuracy when compared to traditional methods through periodic updates
Ren et al. [49]	A network intrusion detection model (ID-RDRL) based on RFE feature extraction and deep Reinforcement Learning	DQN, RFE	The model can smartly choose the best subset of features, eliminate the non-essential ones, and acquire the features through DRL to boost the IDS performance
Lopez-Martin et al. [48]	A novel combination of several DRL algorithms for intrusion detection over a labelled dataset	DQN, DDQN, Policy Gradient, Actor-Critic	The highest performance is achieved for the DDQN algorithm in comparison with others
Alavizadeh et al. [50]	A new generation manner that blends a Q-learning driven RL with a deep feed forward neural network method for network intrusion detection	Deep Q-Learning	The DQL is extremely potent in differentiating between intrusion classes and outclasses other akin ML techniques

From the studies, many models presented high accuracy, precision, recall and F1-scores, usually over 90% and sometimes exceeding 99% on benchmark datasets. Comparative studies demonstrated varying results. DDQN was found superior for supervised adaptations in an instance [48] and SARSA also showed high consistency in another [45]. With lower discount factors mostly, DQL performed well and Actor-Critic was noted to be stable across dynamic parameters [44,50]. In studies where hybrid models combining RL with techniques like Deep AE, Improved Flamingo Search Algorithm (IFSA), Genetic Programming Combiner (GPC) and Voting, highest accuracies were achieved [46,47]. The strong performance of RL in recall highlighted a significant impact on false negatives as according to studies [48]. RL was consistently found to be stronger when it comes to adapting to altering threats when compared to ML. Without updates, traditional ML models were noted to deteriorate over time [45,47,49]. An RL approach was specifically designed to extend model lifespan in a study, achieving reliability even without updates [11]. The challenge of feature drift is addressed with RL-based dynamic feature selection in a study. The study uses a Voting Enhanced DQN Multi-Agent Feature Selection (VE-DQN-MAFS) within Incremental Feature Drift-Aware GPC (IDFA-GPC) which showed strong results in maintaining performance in evolving feature spaces [47]. DRL models are also noted for faster prediction times compared to some complex alternatives like SVM and Radial Basis Function (RBF) kernels in a study [48]. According to studies, transfer learning approaches for updates significantly reduce computational cost and data requirements as DRL training can be intensive. RFE technique also help reduce computational load, and dimensionality when combined with RL, while improving or maintaining accuracy [11,45,49]. Several studies found that a lower discount factor is crucial for better performance when applying DRL to supervised dataset adaptations, emphasising immediate reward over uncorrelated states. The use of epsilon-greedy strategy with decaying epsilon values over training was common in studies [48,49,50]. DAE has been used with DRL to learn feature representations and approximate Q-functions effectively, especially for high-dimensional data [46]. Natural Language Processing (NLP) techniques like textrank and work embeddings were also used for feature extraction from system call logs in host-based IDS in a study, automatically generating detection rules based on extracted keywords, demonstrating feasibility for host-level protection [44].

The studies generally conclude that RL and DRL offer significant potentials for creating more adaptive, robust, and automated cyberattack detection. The ability to learn from interaction, optimise policies over time, and potentially reduce the burden of model updates are key advantages that were highlighted.

### 3.5. Datasets

Training AI-based IDS with a high-quality dataset that also has the appropriate features has great significance on the accuracy of predictions [51,52]. However, only a limited number of datasets in the IDS domain are publicly available. The available datasets found from the literature listed in Table 6. Unlike other fields which have numerous high publicly accessible quality datasets, the IDS domain continues to face this major issue due to privacy and legal concerns. Benchmark datasets, i.e., the most popular datasets in IDS exist and these include KDDcup99, NSL-KDD, ISCX2012, CICIDS2017 and CICIDS. However, these are outdated datasets from decades ago, making them irrelevant in detecting current attack patterns. Benchmarks are classified into static and dynamically generated datasets. Static datasets are not altered after generation, presenting issues of meeting renewable and flexible datasets requirements, while dynamics can be updated over time to match evolving threats [53,54]. Key challenges associated with these datasets include severe class imbalance, limited coverage of certain attack categories, and an emphasis on generic network traffic rather than the unique characteristics of IoT environments, which raises concerns about the generalisability of resulting models [4]. Most organisations do not release the network traffic due to confidentiality issues. Therefore, there is a huge demand for real-time network traffic data [55]. The evolution of available datasets continues to expand in accompaniment to the increase of cyber threats. Recent dataset development efforts increasingly rely on a combination of flow-based and labelled data. Packet capture tools are commonly used to generate new datasets, as they allow researchers to collect either real network traffic or traffic synthetically produced using traffic generators. Following data capture, labelling is typically performed using flow analysis and feature extraction techniques to prepare the data for classification, although the specific tools and workflows are often insufficiently documented or implemented through ad hoc scripts. Despite a general preference for real-world traffic due to its realism, many studies continue to depend on synthetic or semi-realistic datasets, largely because collecting, labelling, and sharing real network traffic remains technically and ethically challenging [51,56,57]. To validate new datasets, they are trained and tested on AI models and the performance is normally compared to benchmark datasets [58]. There are no standard suitable tools to assess the quality of datasets. However, techniques like permutation testing can help evaluate the quality by analysing the correctness, consistency and separability of labelling, ultimately assessing the relationship between observations and labels of the dataset. This shows a clear advantage in assessing the quality on both real and synthetic datasets [59]. A quality dataset should represent all possible scenarios. A large-scale dataset that contains insights from threat intelligence institutions and the community at large can help meet the demands of large, complex real-time threats while preserving privacy [52]. Comparatively, there is a significant difference in feature distributions between synthetic and real-world datasets, raising generalizability concerns. Researchers are therefore encouraged to integrate more recent datasets or to create new ones that better reflect emerging attack behaviours driven by the rapid evolution of cyber threats. Greater collaboration and information sharing between network operators and the research community would also help address confidentiality barriers that limit dataset availability. A key gap in the literature lies in the absence of comprehensive and systematic evaluations across diverse datasets, which currently constrains the generalisability, flexibility, and real-world applicability of many proposed intrusion detection frameworks [4,60]. Researchers are encouraged to generate datasets utilising tools that can inject anomalies, replicate properties or directly capture network traffic, and/or extract packet features to meet specific threat scenarios [54,61].

**Table 6:** Publicly available datasets.

Works	Datasets Used	Year Developed
Alotaibi et al. [2]	UNSW-NB15	2015
Rajathi and Rukmani [5]	NSL-KDD UNSW-NB15 CICIDS2017	2009 2015 2017
Saeed et al. [45]	CIC-Bell-DNS-EXF-2021	2021
Dash et al. [32]	NSL-KDD CICIDS2017 BoT-IoT	2009 2017 2019
Kim et al. [44]	ADFA-LD LID-DS 2021	2013 2021
Shyaa et al. [47]	KDD Cup ’99 CICIDS2017 ISCX2012 HiKARI-2021	1999 2017 2012 2021
Olanrewaju-George and Pranggono [40]	N-BaIoT	2018
Luay et al. [62]	NetFlow datasets version 3	2025
Cao et al. [63]	LUT13	2013
Verma et al. [64]	ROAD CAN	2020
Ullah and Mahmoud [65]	IoTID20	2020

Dataset Taxonomy and Coverage:Intrusion detection benchmarks can be broadly categorised according to their data representation and application domain. Flow-based datasets (e.g., NetFlow-style summaries) capture aggregated traffic statistics, while packet-level datasets preserve fine-grained header and payload information. Domain-specific datasets further include IoT traffic collections, industrial control system traces (e.g., CAN bus data), DNS traffic logs, and cloud network telemetry. These distinctions are important, as model design and feature engineering requirements vary significantly across data modalities.

Data Quality and Temporal Challenges: Beyond structural differences, dataset quality introduces additional methodological considerations. Label noise may arise from automated labelling processes or simulated attack injections, potentially affecting model reliability. Moreover, most benchmark datasets represent static snapshots of network behaviour, limiting their ability to capture concept drift and evolving attacker strategies. The absence of certain attack categories in widely used datasets also constrains model generalisability. Consequently, reported performance results should be interpreted in light of dataset coverage, representativeness, and temporal validity.

### 3.6. Evaluation Metrics

It is imperative to evaluate the performance of ML algorithms with available metrics to ensure the reliability and effectiveness of the algorithms. Choosing the best metric, however, depends on various factors, such as the nature of the dataset, attack type and system requirements. The employment of combined metrics for performance evaluation is always recommended [15,66]. However, there is an absence of standardised evaluation metrics and the lack of consistency restricts the ability to compare and apply IDS results in a broad and unified approach. Although many studies claim high accuracy rates under controlled conditions, it is believed that contexts in the real-world remain ambiguous and lack of standard measures presents issues for comparing and generalising results across different domains [4]. Notable common metrics in most studies include accuracy, precision, recall, F1-score, Area Under the Receiver Operating Characteristic Curve (AUC-ROC), False Negative Rate (FNR) and False Positive Rate (FPR). Variations in these studies include detection rate, specificity, and Matthews Correlation Coefficient (MCC) among others. Overall, generic metrics (accuracy, precision, recall, F1-score) are usually combined for performance evaluation of models in various research [28,67,68]. However, due to significant flaws of these metrics when performing on multi-class classification, new metrics are created and existing ones are modified as additional metrics. The Common Variable Scoring System (CVSS), an open evaluation framework, can enable the provision of a more informed evaluation of the actual performance of ML-based cyberattack detection [69]. The CVSS is calculated based on three metrics: The Base group which represents the inherent attributes of constant vulnerabilities over time, the Temporal group which represents the qualities of dynamic vulnerabilities over time and the Environmental group which reflects the unique vulnerabilities to an environment [70]. Based on the CVSS, metrics like False Alarm Cost (FAC), Miss Cost (MC) and Cyber Informedness (CI), have been introduced. FAC, a generalisation of False Discovery Rate (FDR), implies the negative consequences of an IDS incorrectly identifying an attack, MC, a generalisation of FNR, helps to determine a model’s failure to detect attacks and CI aggregates both FAC and MC to provide an idea about the performance of cyberattack detection [69,71]. There is also a holistic evaluation metric which includes convergence to reflect the time and resources consumed by an FL algorithm, computation efficiency to assess the total time or memory required, fairness for the difference in model accuracy in an FL system and personalisation to evaluate the customisation effectiveness of participants. These metrics are specifically favourable in evaluating FL systems [72].

In multi-class intrusion detection tasks, standard metrics may provide an overly optimistic assessment when class distributions are highly imbalanced. For example, in a dataset containing predominantly benign traffic with only a small proportion of rare but critical attack types (e.g., privilege escalation or data exfiltration), a classifier may achieve high overall accuracy while failing to detect the most consequential attacks. Similarly, micro-averaged precision and F1-scores can be dominated by majority classes, masking poor performance on minority categories. Alternative metrics such as macro F1 attempt to balance class contributions by averaging performance across classes, while weighted F1 incorporates class frequency into the evaluation. The MCC provides a more balanced assessment by considering all elements of the confusion matrix. However, these metrics remain purely statistical and do not reflect the operational severity of different attack types. Incorporating CVSS-informed or cost-sensitive evaluation approaches enables performance assessment that accounts for the relative impact and risk level of detected attacks. In IDS applications, where the consequences of missing a high-severity intrusion differ substantially from misclassifying benign traffic, such risk-aware evaluation may offer a more meaningful measure of practical effectiveness.

It is important to emphasise that reported performance values across different studies should be interpreted within their respective experimental contexts. Variations in dataset composition, class imbalance, preprocessing strategies, and evaluation protocols limit direct numerical comparability. Consequently, performance figures presented in this review serve as contextual indicators rather than standardised benchmarks. A fully normalised cross-study comparison would require unified experimental replication, which is beyond the scope of this survey.

### 3.7. Computational Power

Choosing the appropriate hardware to train and operate AI techniques has a significant impact on the performance and quality of the models. Important hardware requirements for an effective operation include advanced processors like Central Processing Unit (CPU), Graphics Processing Unit (GPU), Tensor Processing Unit (TPU) and Field-Programmable Gate Array (FPGA), sufficient memory and storage [73]. Allocating computational resources for AI applications efficiently is key as AI model workloads differ and hence may require specific resources [74]. Application of an AI technique with insufficient computational resources cannot achieve strict real-time performance. Thankfully, advancements in computational resources are constantly being made. There is a need for effective computational optimisation, however, which can be used to implement AI algorithm in accordance with a specific hardware architecture [75], and there is a rapid escalation in computing needs for AI techniques [76]. This section provides a brief overview of resources normally used for AI-based cyber attack detection.

From the Table 7, the minimum RAM size used in the highlighted research is 8 GB. All computers appear to have high processing units with current operating systems. The computer with the highest computer power had 128 GB of RAM size and with an Ubuntu system. Ideally, FL may require larger storage and memory size and more advanced processors due to its workload. Beyond raw specifications, it is important to consider how different hardware architectures impact the inference speed, energy efficiency, and model scalability of AI-based IDS. For example, Graphics Processing Units (GPUs), such as NVIDIA A100, are highly effective for training Deep Learning models due to their parallel processing capability, but they may not be energy-efficient or cost-effective for real-time or edge deployments. In contrast, Tensor Processing Units (TPUs) offer lower latency and high throughput, making them suitable for large-scale cloud deployments, while Field-Programmable Gate Arrays (FPGAs) and edge accelerators (e.g., Google Coral, Jetson Nano) provide lower power consumption and can be embedded in resource-constrained environments such as IoT networks [16,77]. Comparative studies have shown that model performance can degrade significantly when migrated from high-resource environments (like cloud GPUs) to edge devices if not optimised appropriately. For instance, a ResNet-based IDS model trained on a server with 32 GB RAM and NVIDIA RTX 3090 may experience a 50–70% slowdown in inference time and increased latency when deployed on an 8 GB Jetson Nano unless quantisation or pruning is applied [46,78]. Moreover, some FL models demand consistent memory and network bandwidth allocation which may not be feasible in non-dedicated edge environments due to frequent parameter synchronisation. Hence, hardware-aware model optimisation, such as Neural Architecture Search (NAS) or runtime-specific model conversion (e.g., ONNX, TensorRT), is becoming an essential part of AI pipeline design for Intrusion Detection Systems [46,63]. Finally, the trade-off between real-time detection capability and computational cost must be strategically managed. Systems designed for enterprise-grade cybersecurity can afford high-performance clusters, while IoT and smart-grid environments demand lightweight, efficient models that can execute within strict latency bounds. As AI adoption in IDS grows, the selection of computing platforms and the corresponding optimisation of models to fit those platforms will be a critical determinant of operational success.

**Table 7:** Computing specifications used.

Works	Processor	RAM	Operating System	Model Approach
[2]	6th Gen Intel Core i5 @ 3.30 GHz	8 GB DDR4 @ 2401 MHz	Windows 10 version 1903	Hybrid system
[5]	Intel Core i5-10210U @ 1.60 GHz (up to 2.11 GHz)	8 GB	64-bit Windows on x64-based processor	Hybrid system
[29]	13th Gen Intel Core i5-1345U @ 1.60 GHz, 8-core CPU, 8-core GPU	16 GB		Anomaly-based
[46]	Intel Core i5-3470 @ 3.20 GHz, 4 cores, 4 logical Pro	8 GB	Windows 10 Pro version 10.0.19044	Reinforcement Learning
[41]	Intel Xeon NVIDIA A100 GPU	128 GB	Ubuntu 20.04	Federated Learning
[8]	Intel Core i7/i9 or AMD Ryzen 7/9	16 GB or higher		ML and DL
[79]	AMD Ryzen 7 4800H	16 GB		Federated learning
[77]	Intel Core i5-10400F GPU Nvidia GTX 1650 OC-4G	16 GB	Ubuntu 20.04.4 LTS	DT, RF, KNN, MLP and BernoulliNB

While many surveyed studies report high-performance computing environments for model training and evaluation, real-world deployment often occurs under significantly constrained conditions. AI-driven IDS must operate across heterogeneous environments ranging from low-power IoT devices to enterprise-scale cloud infrastructures. Therefore, computational feasibility, latency, and energy efficiency become critical factors.

IoT and Embedded Devices: In IoT environments, devices typically possess limited CPU capability (e.g., ARM Cortex processors), restricted memory (often <1 GB RAM), and strict energy budgets. In such contexts, lightweight ML models (e.g., Random Forest, SVM with reduced feature sets) are preferable due to lower computational overhead and faster inference time. Deep Learning models, unless compressed via pruning or quantization, may introduce excessive latency and power consumption.

Edge Computing Gateways: Edge nodes (e.g., fog servers or smart gateways) offer moderate computational resources and can support compact Deep Learning models such as CNNs or LSTMs with optimized architectures. Federated Learning becomes particularly attractive in these environments, as it allows collaborative anomaly detection while preserving data privacy and reducing centralized data transfer.

Enterprise Servers and Security Operation Centres (SOC): In enterprise environments, GPU-enabled servers allow deployment of Deep Learning, generative models, and Reinforcement Learning agents for adaptive threat detection. Here, higher computational cost may be acceptable in exchange for improved zero-day detection and adaptive defence capabilities.

Cloud-Based Infrastructure: Cloud platforms provide scalable computing power suitable for training large Deep Learning and generative AI models. However, trade-offs include higher operational costs, potential data privacy concerns, and increased latency due to data transmission from edge devices.

In real-world deployments, enhancing detection accuracy often increases computational demand. Advanced Deep Learning and generative models can detect complex and previously unseen attacks, but they require significant processing power, memory, and training data. In contrast, lightweight Machine Learning techniques offer faster inference and lower resource consumption, making them suitable for constrained environments, though they may struggle with highly sophisticated threats. Federated and Reinforcement Learning approaches introduce additional overhead in communication or training time. Therefore, selecting an appropriate model requires balancing detection capability with hardware limitations, latency requirements, and operational constraints.

Deployment strategies should align with the available infrastructure and risk profile. In IoT and embedded environments, lightweight ML or compressed DL models are preferable due to limited computational and energy resources. Distributed enterprise systems may benefit from Federated Learning to preserve data privacy while enabling collaborative detection. For adaptive defence in dynamic threat landscapes, Reinforcement Learning can provide flexible response mechanisms. In high-security critical systems, hybrid or ensemble approaches can enhance robustness while maintaining acceptable performance.

### 3.8. Explainable AI in Cyberattack Detection

AI models, particularly Deep Learning and Reinforcement Learning techniques, have demonstrated exceptional performance in detecting cyberattacks. However, their inherent complexity and “black-box” nature often negatively influence transparency and trustworthiness. This need has driven the adoption of Explainable Artificial Intelligence (XAI) as a critical element of AI-based Intrusion Detection Systems, enabling greater interpretability, trust, and auditability [80]. XAI focuses on making model decisions understandable to human analysts while preserving predictive performance. Commonly used approaches, such as Shapley Additive Explanations (SHAP) and Local Interpretable Model-agnostic Explanations (LIME), help reveal the influence of individual input features on detection outcomes, thereby supporting transparency and informed decision-making [81,82].

Table 8 summarises the XAI methods that align best with different IDS design goals. Mallampati et al. [81] proposed a transparent XAI framework using SHAP in benchmark datasets such as NSL-KDD and CICIDS-2017, which improved human interpretability and trust in IDS models. A comprehensive survey by Neupane et al. [82] emphasised the importance of integrating interpretability in IDS, their work revealed that many deep models are difficult for analysts to understand and validate even when highly accurate, especially during false positive analysis or high-severity threat response. Similarly, Marino et al. [80] introduced an adversarial approach to XAI for IDS, where adversarial examples helped to interpret model sensitivity to slight perturbations in inputs, revealing underlying vulnerabilities and hidden decision logic. Further work by Ables et al. [83] demonstrated how self-organising maps (SOM) can be used to visualise and explain complex data distributions in intrusion detection. Their method provided both local and global explanations, offering clarity on how certain inputs influence anomaly scores across the datasets. In IoT and host-based systems, Mane and Rao (2021) and Islam et al. (2019) showcased how domain knowledge combined with XAI techniques could significantly improve decision transparency. By integrating contextual reasoning, such as using SHAP along with domain-specific attack semantics—they enabled interpretable models that align with established cybersecurity standards (e.g., CIA Triad: Confidentiality, Integrity, and availability) [84,85]. The application of XAI not only supports regulatory compliance and ethical transparency, but also plays a critical role in identifying bias, adversarial susceptibility, and model weaknesses. These are often hidden within black-box AI models. As AI becomes increasingly integrated into critical infrastructure, the demand for interpretable and human-centred AI will only grow. Incorporating XAI into cyberattack detection models enhances their practical utility, security practitioner understandability, promotes trust, and aids in incident analysis. Future research directions include combining XAI with adversarial robustness, developing visual analytics dashboards, and advancing explanation-aware training techniques.

**Table 8:** Explainable AI techniques used in cyberattack detection.

Works	XAI Technique	Explanation Type	Compatible Models	Key Contribution/Strength
Mallampati et al. [81]	SHAP (Shapley Additive Explanations)	Feature importance (global and local)	DL (ANN, LSTM), RF	Improved interpretability on NSL-KDD and CICIDS2017
Neupane et al. [82]	SHAP, LIME, Anchors (XAI Taxonomy)	Post-hoc, model-agnostic	DL, SVM	Emphasised interpretability needs for high-risk threats
Marino et al. [80]	Adversarial Example-based Explanation	Sensitivity to input perturbations	CNN, DNN	Revealed model weaknesses using adversarial attacks
Ables et al. [83]	Self-Organising Maps (SOM)	Visual and cluster-based explanation	ML, unsupervised models	Visualised anomaly clusters and decision boundaries
Mane and Rao [84]	SHAP + domain semantics	Hybrid (XAI + domain logic)	IoT, embedded DL models	Mapped model outputs to CIA triad for IoT threats
Islam et al. [85]	Context-aware explanation with SHAP + knowledge base	Rule-based with semantic reasoning	Host-based IDS	Mapped anomalies to semantic attack types for transparency

It is important to distinguish between the predictive model and the explanation mechanism. Post hoc explainability techniques such as SHAP and LIME typically operate after model inference and therefore do not directly modify the trained classifier or degrade its detection accuracy. However, generating explanations, particularly for complex Deep Learning architectures can introduce additional computational overhead. In high-throughput or real-time intrusion detection environments, this may affect response latency, requiring selective or on-demand explanation generation rather than continuous interpretation for every prediction.

### 3.9. Emerging AI Techniques

As the cybersecurity landscape continues to evolve, novel AI techniques are emerging to overcome the limitations of traditional ML, DL, FL and RL in detecting cyberattacks. This section highlights three promising directions: (i) generative AI, (ii) neuro-symbolic AI, and (iii) swarm intelligence with bio-inspired algorithms and their potential applications in strengthening IDS. These techniques offer innovative solutions to challenges such as data scarcity, interpretability, and optimisation in complex, dynamic environments.

#### 3.9.1. Generative AI for Cyberattack Detection

Generative AI, encompassing models like GANs and LLMs, has shown significant potential in cybersecurity by addressing data scarcity and enhancing detection capabilities. GANs, as demonstrated in the paper [86], are used to generate synthetic attack patterns, which are critical for training IDS in scenarios where real-world attack data is limited or sensitive. For instance, DCGANs can create realistic network traffic patterns to simulate advanced persistent threats (APTs) or zero-day attacks, enabling robust model training [86]. Recent studies have extended this approach by using diffusion models, which offer improved stability over GANs for generating high-fidelity synthetic datasets [87]. These synthetic datasets help address class imbalance issues, a persistent challenge in cyberattack detection, by augmenting minority class samples, similar to the SMOTE as discussed in Section 3.7 [48].

Moreover, multimodal LLMs are increasingly applied to process diverse data types, such as network logs, system calls, and threat intelligence reports written in natural language. These models can extract contextual patterns from heterogeneous data sources, improving the detection of sophisticated attacks like phishing or ransomware [88]. For example, a recent study proposed a multimodal LLM-based IDS that integrates network packet data with textual threat intelligence, achieving F1-scores above 97% on the CICIDS2017 dataset [88]. However, generative AI models face challenges including high computational costs and the risk of generating adversarial examples that could be exploited by attackers, necessitating robust defences like adversarial training.

#### 3.9.2. Neuro-Symbolic AI

Neuro-symbolic AI brings together the pattern-learning strengths of neural networks and the logical reasoning capabilities of symbolic systems, providing a promising pathway to enhance both the interpretability and robustness of IDS. Unlike traditional DL models which are often criticised for their “black box” nature, neuro-symbolic AI integrates domain knowledge, such as cybersecurity rules or attack signatures, with data-driven learning. This hybrid approach enhances the detection of complex attacks by reasoning over structured knowledge while leveraging neural networks for feature extraction [89]. For instance, a neuro-symbolic IDS can use symbolic rules to identify known attack patterns (e.g., SQL injection) while employing neural networks to detect anomalous behaviours in network traffic.

A recent study proposed a neuro-symbolic framework for IDS that combines a CNN for feature extraction with a rule-based reasoning module to explain detection decisions, achieving a precision of 96% on the NSL-KDD dataset [89]. This framework addresses the interpretability challenge highlighted in Section 6.1 by providing human-understandable explanations, aligning with the XAI goals discussed in Section 3.7 [34,48]. Neuro-symbolic AI requires careful integration of symbolic and neural components, as mismatches can lead to reduced accuracy or increased computational complexity. Future research should focus on optimising these integrations and scaling neuro-symbolic models for real-time IDS applications.

#### 3.9.3. Swarm Intelligence and Bio-Inspired Optimisation Techniques

Swarm intelligence and bio-inspired optimisation techniques, such as PSO, JAYA, and COA, are increasingly used for optimising IDS performance, as briefly mentioned in Section 3.2. These algorithms mimic natural processes, such as the collective behaviour of swarms or biological evolution, to optimise feature selection, hyperparameter tuning, and model performance in cyberattack detection. For instance, PSO has been applied to identify the most informative features within high-dimensional network traffic data, thereby reducing computational complexity while enhancing detection performance [17]. Recent work has shown that integrating PSO with a Random Forest classifier can significantly improve intrusion detection outcomes, achieving a detection accuracy of 98.5% on the CICIDS2017 dataset through effective feature selection [90].

Other bio-inspired algorithms, such as Artificial Bee Colony (ABC) and Ant Colony Optimisation (ACO) optimisation, are gaining traction for their ability to solve complex optimisation problems (e.g., minimizing false alarms, detection accuracy and speed) in IDS. ABC has been applied to optimise the weights of neural networks in IDS, improving convergence speed and detection accuracy for IoT environments [91]. Similarly, ACO has been used for dynamic feature selection in real-time IDS, adapting to evolving attack patterns [91]. These algorithms offer advantages over traditional optimisation techniques by providing robust, scalable solutions for resource-constrained environments, aligning with the lightweight AI goals discussed in Section 4. However, challenges include the computational cost of iterative optimisation and the need for careful parameter tuning to avoid local optima. Future research should explore hybrid bio-inspired approaches, combining multiple algorithms to enhance IDS performance.

To facilitate a deeper understanding of the potential of generative AI, neuro-symbolic AI, and swarm intelligence and bio-inspired algorithms in cyberattack detection, Table 9 provides a comparative analysis of their application, strength and limitations.

**Table 9:** Comparison of emerging AI techniques for cyberattack detection.

Technique	Primary Application	Strengths	Challenges	Computational Requirements
Generative AI (e.g., GANs, LLMs)	Synthetic data generation, multimodal threat detection	Addresses data scarcity, handles diverse data types, high accuracy [88]	High computational cost, risk of adversarial examples [87]	High (GPU/TPU, >16 GB RAM)
Neuro-symbolic AI	Interpretable IDS, rule-based anomaly detection	Combines reasoning and learning, enhances interpretability [89]	Complex integration, scalability issues for real-time applications	Moderate (8–16 GB RAM)
Swarm intelligence (e.g., PSO, ABC)	Feature selection, hyperparameter optimisation	Scalable, efficient for resource-constrained environments [90,91]	Iterative optimisation, parameter tuning complexity	Low (4–8 GB RAM)

### 3.10. Systems-Level Integration of FL, RL, and XAI

In practical deployments, FL, RL, and XAI may operate at complementary layers within an intrusion detection architecture. Federated Learning can enable distributed model training across edge nodes while preserving data locality and privacy. Reinforcement Learning may function at the policy layer, adapting detection thresholds or response strategies based on observed network dynamics. Explainable AI techniques can provide interpretability at the decision layer, supporting operator trust and regulatory compliance. However, integrating these components introduces system-level considerations including communication latency, orchestration overhead, synchronisation frequency, and response timing constraints. Balancing adaptive intelligence with real-time performance remains an open research challenge in large-scale cyber-physical systems.

## 4. Lightweight AI for Cyberattack Detection

**Figure 3:**
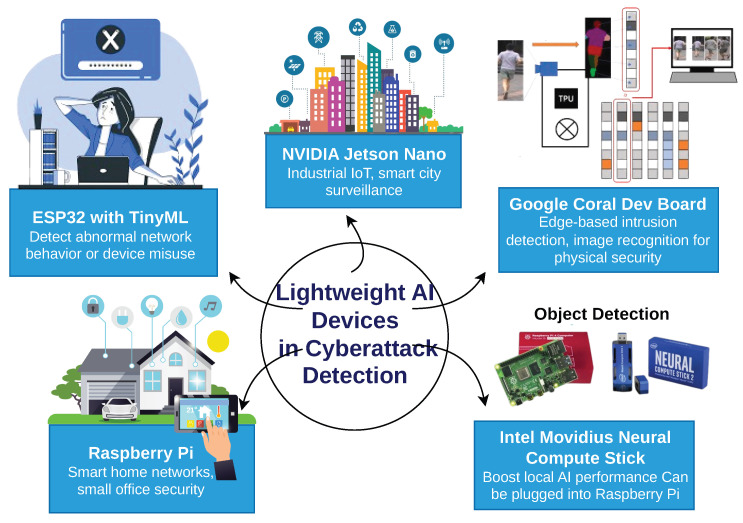
Smart intelligence in lightweight devices.

Traditional AI-based approaches have demonstrated strong potential in attack detection, yet their high computational requirements often limit their suitability for deployment in resource-constrained environments. Hence, studies propose computational efficient and cost-effective lightweight techniques for cyberattack detection [92]. Lightweight AI models are simplified ML models and systems optimised to run efficiently on devices with limited resources. The ability of lightweight models to deliver AI capabilities on devices without having to rely heavily on vast resources helps reduce latency and improve privacy, making it an important application in cyberattack detection. Known strategies for developing lightweight models are model compression techniques, knowledge distillation and other optimisation techniques. Compression techniques include pruning; where less relevant parameters are removed from models, and quantisation; which reduces the computational overhead and memory of models and speeds up inferences. In knowledge distillation, the knowledge of typically large and complex models is transferred to develop smaller ones. Simplifying the architecture of AI models, transfer learning and ensembles focusing on smaller, efficient models to improve performance are some of the optimisation techniques [93]. Based on this context, various studies have been conducted exploring various approaches to assess the performance of lightweight models in cyberattack detection in hopes for high precision of detection while providing resource-preserving solutions [94,95,96]. The use of a learning RNN to build lightweight detector for certain types of attacks on IoT systems in one instance concluded high detection rates with few false alarm rates [97]. A meta-learning deployment using lightweight attack detection models also presented consistently stable high accuracy with low false positive rates across different datasets, while maintaining reasonable inference times [98]. Lightweight DL model for efficient attack detection in cloud computing environments has also been explored, achieving high training and testing accuracies, while addressing resource constraints. The model was also concluded to be reliable for scenarios that require swift and dependable attack detection [99]. Application of lightweight AI in FL are also being explored to address challenges with regard to privacy, system complexity and scalability. Harnessing FL and structured model pruning for cyberattack detection highlights the potential of lightweight AI to enhance security while addressing these challenges and resource constraints in distributed environments. The results in these endeavours indicate acceleration in training time compared to traditional methods while maintaining high detection accuracies [78,100]. Lightweight models must balance accuracy and efficiency, often requiring trade-offs in complexity to fit resource constraints. Simplifying models can reduce the ability to process huge datasets or execute complex tasks, potentially leading to less precise or personalised outcomes. Techniques likes model compression and FL can be essential when utilised to maintain performance while reducing direct device computation. All lightweight models must be scalable across different platforms demanding optimisation tailored to hardware, operating systems and user interfaces. Developing more efficient algorithms that can produce high performance with limited resource usage is still advised. Advancements are required in techniques like model pruning, quantisation and neural architecture search to help enhance speed and efficiency in lightweight AI [93,101]. Figure 3 illustrates the role of lightweight AI-enabled devices in supporting cyberattack detection across distributed and resource-constrained environments. The diagram highlights representative platforms, including ESP32 with TinyML for abnormal network behaviour detection, Raspberry Pi for smart home and small office security, NVIDIA Jetson Nano for industrial IoT and smart city surveillance, Google Coral Dev Board for edge-based intrusion and image analysis, and the Intel Movidius Neural Compute Stick for accelerating local inference. Collectively, these devices demonstrate how edge computing and compact AI hardware can enable real-time monitoring, anomaly detection, and object recognition without reliance on centralised cloud infrastructure. Table 10 presents a summary of related works in lightweight techniques in cyberattack detection.

**Table 10:** Summary of lightweight techniques for cyberattack detection in other research.

Works	Proposed Methodology	Lightweight Technique	Performance
Bouayad et al. [78]	The major intention was the substitution of already existing methods with the Lightweight-Fed-NIDS which would be much cheaper and safer in terms of data privacy	Smartly executed pruning	The method reaches up to 3× faster training time when compared with the traditional unpruned FL models and still has a 99% detection rate.
Soomro et al. [100]	An innovative lightweight federated deep Intrusion Detection System that leverages CNNs, LSTMs, and MLPs within a Federated Learning framework to preserve data privacy, while reducing system complexity and improving scalability	Tailored optimization strategy	The proposed approach demonstrates strong efficiency across diverse edge devices, achieving an accuracy of 98.68%, a precision of 98.78%, a recall of 98.64%, and an F1-score of 99.05%.
Ismail et al. [94]	A comparative study and performance analysis of several ML classification techniques, with a focus on supervised methods to identify the lightweight model for cyberattack detection suitable for deployment in resource-constrained IIoT environment	Unspecified	RF, Bagging, Stacking, and Catboost are seen to perform well in terms most of the metrics; however, Stacking model achieves the best accuracy, recall, micro F1-score, macro F1-score, and MCC, and Catboost has the highest Precision
Otokwala [92]	An Optimised Common Feature Selection and Deep Autoencoder (OCFSDA) approach for lightweight intrusion detection, designed to be computationally efficient and cost-effective for IoT environments	Shallow Deep Learning	The model achieved high detection accuracy across both datasets (99% and 97%), while significantly reducing execution time (0.30 s and 0.12 s) and maintaining minimal memory usage of approximately 2 KB
Tadesse and Choi [101]	A novel approach that employs the Short-Time Fourier Transform (STFT) to design an anomaly detection system for intrusion detection, using a lightweight convolutional neural network to classify denial-of-service and distributed denial-of-service attacks	Custom technique	The model attained high detection accuracy while maintaining notably low false alarm rates, along with strong specificity and sensitivity
Soe et al. [95]	A lightweight ML-based IDS using a new correlated-set thresholding on gain-ratio (CST-GR) algorithm for feature selection	Pruning	The system effectively selects new features with almost no sacrifice on detection performance
Chaganti [102]	A three-layer security framework that combines AI-based intrusion detection, blockchain for decentralised trust management, and edge computing for efficient resource utilisation	Mathematical optimisation	The proposed framework provides a scalable, adaptive, and efficient IoT security solution with 94.78% accuracy, making it suitable for resource-constrained environments and real-time applications
Singamsetty [103]	An enhanced cyberattack detection system for edge-based IoT networks, employing a fuzzy-based Siberian Tiger Optimisation (FSTO) algorithm alongside advanced Machine Learning methods	Bayesian optimisation, Quantisation	The framework shows significant improvements across several performance indicators including accuracy, precision, recall, F1-score, and Area Under the Curve (AUC)

## 5. Quantum Computing for Cyberattack Detection

**Figure 4:**
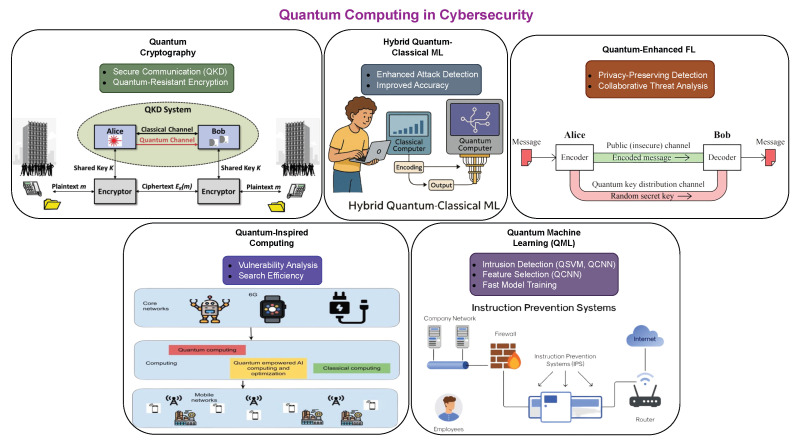
Application of quantum computing in cybersecurity.

The emergence of quantum computing represents a significant shift in the cybersecurity landscape, offering new opportunities to strengthen organisational security. As illustrated in Figure 4, quantum computing has the potential to support cyberattack detection by addressing problems that are difficult to solve using classical approaches. By exploiting the fundamental principles of quantum mechanics, quantum computing enables substantially enhanced computational capabilities, opening the door to breakthroughs in complex problem-solving. These advances are expected to drive transformative progress in both computing and communication technologies, with important implications for next-generation cybersecurity systems [104,105]. Quantum ML, an integration of quantum computing with ML approaches, is being applied in various security domains to handle challenges encountered, especially on big data. The application of quantum computing to traditional ML and DL algorithms have been proven to significantly outperform traditional models in cyberattack detection [106,107].

Platforms and frameworks to enable the implementation of quantum computing exist, of which, the Qiskit platform and the TensorFlow Quantum framework are considered to be the some of the most promising ones. QML-based intrusion detection is found to make more effective protection compared to traditional ML approaches, especially on large-scale network with a vast amount of security-relevant data. Methods like QSVM and QCNN have demonstrated a clear advantage of the quantum-based approach in terms of model accuracy, while reducing training time drastically. Compared against each other, QCNN is considered more promising as it selects the most significant features with high probabilities although QSVM is faster. Future targets to be explored is optimisation of the quantum algorithms and parallelisation schemes for the fast training of QML models [106,108]. Hybrid quantum ML has also been studied, in which quantum ML is combined with a classical ML model to improve attack detection. Although, quantum ML is shown to perform better than traditional ML models, the hybrid system is also proven to perform better than both methods [109]. Applications in FL domains are also being developed with significant results in terms of detection accuracy [110]. Quantum cryptography is important in mitigating threats from quantum computing, but it also introduces a range of new challenges including inaccurate performance in high-traffic environments and increased vulnerabilities to attacks provided by heightened computational demands. Migrating to these advanced algorithms is vital to defend against the enhanced capabilities of threatening quantum computers. Quantum attackers constantly leverage on sophisticated techniques to search for weaknesses on quantum and classical computing frameworks [104]. Thus, researchers are exploring quantum inspired computing and optimisation methods to take advantage of quantum advantages over conventional hardware, improving search efficiency, and reducing computational costs [105]. There is also the need for proactive measures to address the potential risks and uncertainties the impact of quantum computing on cybersecurity comes with. A proposal to explore and analyse new quantum learning models, including hybrid and fully quantum approaches, tailored to the field of cyberattack detection is advised. These models could take advantage of the unique strengths of quantum computing to improve detection accuracy and efficiency. Today’s quantum computers can also be utilised further in practical implementations, taking into account existing limitations. This can involve deploying models on real quantum devices and assessing their feasibility, performance, and potential for extension in real network environments [111]. A summary of the applications of quantum technique in cyberattack detection is summarized in Table 11.

### 5.1. Quantum and Hybrid Quantum–Classical Machine Learning (QML and HQML)

Quantum Machine Learning (QML) applies quantum computing principles to enhance Machine Learning models, offering potential advantages in processing speed, accuracy, and efficiency. Key quantum models include quantum support vector machines (QSVM), quantum convolutional neural networks (QCNN), quantum k-nearest neighbours (QkNN) and quantum generative adversarial networks (QGAN) [106,112]. Recently, hybrid quantum–classical approaches (HQML) have emerged as a practical compromise between full quantum systems and classical models. In such systems, data preprocessing and feature extraction are performed using classical methods (e.g., principal component analysis, CNNs), while the classification layer or part of the learning model is quantum-based, such as a Variational Quantum Classifier (VQC). This setup makes efficient use of existing quantum hardware and reduces overhead related to qubit limitations. For instance, the QML-IDS framework integrates a hybrid model that combines classical feature engineering with quantum circuit-based classification. When evaluated on benchmark intrusion detection datasets, including CICIDS2017 and UNSW-NB15, this approach achieved higher detection accuracy and faster inference times than traditional models, underscoring the practical potential of HQML for real-world cyberattack detection systems [86,106].

In practice, HQML systems can be simulated on platforms like IBM Qiskit, PennyLane, or TensorFlow Quantum. These allow researchers to test quantum components while deferring deployment on physical quantum hardware until systems scale up.

### 5.2. Quantum-Enhanced Federated Learning (QFL)

FL enables distributed model training without sharing raw data, which is ideal for sensitive environments like cybersecurity. Quantum-Enhanced Federated Learning (QFL) takes this a step further by incorporating quantum computation into the FL process—either at the local client level or during model aggregation [115]. In QFL systems, each edge node trains a quantum or hybrid model on local data, and shares only encrypted model weights or gradients. These are then aggregated by a central server to update the global model. This enhances privacy and robustness, particularly when dealing with high-dimensional data or latency-sensitive applications like real-time intrusion detection. Recent research showed that using quantum inspired federated aggregation techniques (QIFA) improves convergence and model performance [116]. Studies in datasets such as NSL-KDD and UNSW-NB15 reported F1 scores above 98% using hybrid QFL models [115]. These systems are particularly useful for securing smart cities, IoT networks, and industrial control systems, where device heterogeneity and privacy are critical.

### 5.3. Quantum Cryptography and Post-Quantum Security

Quantum cryptography offers unbreakable encryption through quantum mechanical principles. A widely used techniques is Quantum Key Distribution (QKD), specifically the BB84 protocol, which ensures secure key exchange by detecting any eavesdropping during transmission. When combined with a one-time pad encryption, QKD enables information-theoretically secure communication between IDS agents, edge nodes, and central systems [117]. However, implementing QKD requires specialised photonic hardware and trusted relay stations, which makes its widespread deployment challenging. As a scalable alternative, Post-Quantum Cryptography (PQC) algorithms, which are resistant to quantum attacks but executable on classical systems, are being rapidly adopted. Many governments and large organisations are preparing for “Q-Day”, the point at which quantum computers could break existing public-key encryption schemes [118]. In IDS infrastructures, quantum cryptography can be used to secure:

- Communication between distributed intrusion sensors and central analysers.
- Updates of the Federated Learning model in QFL.
- Real-time alert dissemination and control instructions across the network.

While these techniques offer a powerful toolkit for secure IDS communication, there remain significant limitations that affect current implementations. These challenges mean that, at present, quantum cryptography is more suited to controlled environments than to fully decentralised, large-scale IDS deployments.

### 5.4. Limitations, and Future Outlook of Quantum Computing in IDS

Despite its promise, quantum-enhanced intrusion detection faces several current constraints:

• Hardware Limitations: Current generation Noisy Intermediate-Scale Quantum (NISQ) computing devices typically support fewer than 100 qubits and are prone to noise, limiting usable circuit depth and stability.
• Encoding Overhead: Translating classical data (e.g., network packets) into quantum representations (via amplitude or angle encoding) introduces significant overhead and may negate speed advantages.
• Simulation Dependence: Most proposed models are tested on quantum simulators rather than real devices, raising questions about real-world scalability and deployment.
• Lack of Demonstrated Quantum Advantage: In many cases, performance improvements of quantum models over classical Deep Learning are modest or unverified under production conditions [86,106].

Nevertheless, the future potential of this technique is substantial, especially in environments that require high scalability, privacy, and computational speed. Key directions include:

• Scalable QFL: Fully distributed, privacy-aware detection across 6G-enabled IoT networks.
• Quantum Feature Optimisation: Using quantum annealing or QPSO for feature selection and parameter tuning in IDS.
• Edge-QML Convergence: Integration of QML, FL, and post-quantum cryptography with Edge AI in real-time, latency-sensitive environments.

The robust real-world deployment of quantum-based IDS systems is expected to mature in 5–10 years, coincident with advances in quantum error correction, middleware toolkit, and quantum–classical orchestration platforms.

## 6. Research Challenges and Directions

The application of AI in cyberattack detection has significantly advanced IDS, but several challenges limit its full potential. This section outlines key obstacles in interpretability, computational costs, data quality, real-time application, privacy, and emerging techniques, proposing actionable future research directions to address these issues and enhance AI-driven cybersecurity.

### 6.1. Interpretability Challenges

Despite DL and RL models achieving high accuracy, these models often suffer from a “black-box” nature, reducing trust in critical cybersecurity applications. XAI techniques like SHAP and LIME have been proposed to enhance transparency, but their integration remains limited. Future direction could develop advanced XAI frameworks such as visual analytics dashboards and explanation-aware training to improve model interpretability while maintaining performance.

### 6.2. Computational Resource Constraints

Training complex DL and RL models or optimising hyperparameters (e.g., using Randomised Search) requires significant computational resources, often necessitating GPUs with 16 GB+ memory and hours of training time on datasets like CICIDS2017. Lightweight AI and quantum models also face resource challenges in constrained environments. Future direction could optimise algorithms for specific hardware architectures, advance model compression techniques (e.g., pruning, quantisation), and explore more on hybrid quantum–classical models to reduce computational overhead.

### 6.3. Data Quality and Availability

The performance of the AI model is heavily dependent on high-quality, representative datasets. Benchmark datasets like KDDCup’99 and NSL-KDD, with attack-to-normal traffic ratios often below 1:10, are outdated and fail to capture modern attack patterns. Imbalanced datasets exacerbate detection challenges, requiring techniques like SMOTE. Synthetic datasets should be developed using GANs to simulate evolving threats and encourage network operators to share anonymised real-time data to address confidentiality concerns.

### 6.4. Real-Time Application Issues

Achieving strict real-time performance in resource-constrained environments (e.g., IoT, edge devices) remains challenging due to latency and processing limitations. Lightweight AI models show promise but struggle to balance accuracy and efficiency. Future research could investigate edge-cloud hybrid architectures and asynchronous FL protocols to enable scalable, low-latency IDS deployment in diverse network environments.

### 6.5. Privacy and Ethical Concerns

Although FL mitigates direct data sharing, it still introduces privacy risks due to non-IID data distributions and the exchange of model parameters. Moreover, ethical aspects such as model bias, fairness, and potentially unequal detection outcomes remain insufficiently explored in current FL research. Implementing cryptographic methods (e.g., Quantum Key Distribution) and blockchain-based smart contracts are worthy of consideration for secure, decentralised FL updates. Developing fairness-aware AI models and ethical guidelines to ensure equitable cybersecurity solutions could also be future research directions.

### 6.6. Emerging AI and Quantum Computing Challenges

Emerging AI techniques, such as generative AI, neuro-symbolic AI, and swarm intelligence, face challenges like high computational costs, integration complexity, and limited scalability in dynamic datasets. Quantum computing, despite its potential, is constrained by NISQ device limitations (e.g., <100 qubits, noise) and encoding overhead for classical data. Future research could investigate the optimisation of generative AI through adversarial training to enable scalable neuro-symbolic frameworks and to advance swarm-based algorithms for robust feature selection. In addition, evaluating quantum models on real quantum hardware and exploring post-quantum cryptographic techniques for secure IDS communication would help bridge the gap between theoretical advances and practical deployment.

Beyond individual model performance, bias and fairness considerations extend to the broader intrusion detection research landscape. Many studies rely heavily on a limited set of benchmark datasets, which may not accurately represent diverse network conditions, organisational contexts, or emerging attack behaviours. Class imbalance, synthetic traffic generation, and selective reporting of favourable metrics can introduce systemic bias in performance claims. Furthermore, comparisons across studies are often affected by inconsistent preprocessing steps, evaluation splits, and experimental configurations. Addressing these issues requires transparent reporting, balanced dataset selection, consistent benchmarking practices, and critical interpretation of results. Incorporating fairness-aware evaluation and acknowledging dataset limitations can improve the reliability and generalizability of AI-based IDS research. The summary of the research challenges presented in Table 12 includes the future directions.

To address the identified challenges, future research should prioritise the development of hybrid IDS models that integrate the complementary strengths of ML, DL, RL, and emerging AI techniques to achieve greater robustness and adaptability. At the system level, incorporating blockchain technologies can enable secure and automated auditing within Federated Learning environments, thereby reducing coordination and trust management overhead. Further work is also needed on asynchronous FL protocols that can effectively accommodate heterogeneous clients with varying computational capabilities, network conditions, and availability. In parallel, advancing hybrid quantum–classical models alongside post-quantum cryptographic mechanisms will be essential to safeguarding IDS infrastructures against emerging quantum-enabled threats. Finally, establishing clear ethical AI guidelines is crucial to address issues of bias, fairness, transparency, and broader societal impact in AI-driven cybersecurity systems.

## 7. Conclusions

This review has examined the evolving role of Artificial Intelligence in cyberattack detection, highlighting how data-driven techniques are reshaping IDS in response to increasingly complex and adaptive threats. By analysing developments across ML, DL, FL, and RL, the study shows that AI-driven IDS can move beyond rigid signature-based approaches and offer more adaptive, accurate, and scalable protection. At the same time, the review makes clear that technical progress alone is not sufficient. Persistent challenges related to data quality, computational cost, real-time deployment, privacy preservation, interpretability, and ethical considerations continue to limit practical adoption, particularly in resource-constrained and safety-critical environments. Emerging directions such as lightweight AI, Explainable AI, and hybrid quantum–classical techniques offer promising pathways, but many of these remain at an early stage of maturity. Overall, the findings suggest that future IDS research must take a more holistic view, balancing detection performance with trust, fairness, efficiency, and deployability. By integrating hybrid AI models, robust evaluation practices, privacy-aware learning, and responsible design principles, the cybersecurity community can move closer to building Intrusion Detection Systems that are not only intelligent, but also reliable and suitable for real-world use.

## Figures and Tables

**Table 2:** Structured Comparison of AI paradigms for cyberattack detection.

Method	Attack Suitability	Zero-Day Detection	Computational Requirement	Training Speed/Complexity	Data Volume Requirement	Interpretability
Machine Learning (ML)	Signature-based, anomaly-based	Moderate (depends on feature design)	Low–Moderate	Fast to moderate	Moderate	High (especially RF, DT)
Deep Learning (DL)	Anomaly-based, complex attack patterns	High	High (GPU required)	Slow (hyperparameter sensitive)	Large datasets required	Low (black-box nature)
Reinforcement Learning (RL)	Adaptive defence, dynamic threats	High (adaptive learning)	High	Slow convergence; interaction-based	Large interaction data	Low–Moderate
Federated Learning (FL)	Distributed anomaly detection	High (collaborative learning)	Moderate–High (communication overhead)	Moderate; depends on aggregation rounds	Distributed local datasets	Moderate
Generative AI	Data augmentation, synthetic attack generation	High (simulated zero-day)	Very High	High training cost	Very large datasets	Low
Neuro-symbolic AI	Hybrid rule + anomaly detection	High (rule + pattern reasoning)	Moderate	Moderate	Moderate	High (logical reasoning layer)
Quantum/Hybrid QML	High-dimensional anomaly detection	Promising (theoretical advantage)	Very High (quantum hardware/ simulation)	Experimental stage	Moderate	Low–Moderate

**Table 11:** Summary of quantum techniques for cyberattack detection in other research.

Works	Proposed Methodology	Quantum Technique	Performance
Shen et al. [105]	An IDS building algorithm using a quantum-inspired computing (QIC) approach	A new Global-best guided quantum-inspired tabu search (GQTS) algorithm	High accuracy on two benchmarks with lower floating-point operations (FLOPs) compared to most ML methods
Salvakkam et al. [107]	To identify intrusions through EICDL (Ensemble Intrusion Detection Model for Cloud Computing Using Deep Learning)	Not mentioned	Existing ML models are outperformed by the new model which detects attacks and intrusions with a recall rate of 92.14%
Subramanian and Chinnadurai [110]	A spatio-temporal attention network (STAN) and a quantum-inspired federated averaging (QIFA) optimization technique to detect cyberattacks, integrating a hybrid FL model	Quantum superposition	Outperformed traditional CNN, LSTM, RNN and FL models in anomaly detection with a maximum precision of 98.2%, recall of 98.5%, f1-score of 98.35%, specificity of 98.2% and accuracy of 98.34%
Islam et al. [109]	An amplitude shift cyberattack detection on a dataset from an in-vehicle controller area network via a hybrid quantum–classical NN was developed	Hybrid quantum–classical technique, Quantum encoding	Detection of the attack with 94% accuracy is achieved which is more than that of long short-term memory NN (88%) or quantum NN alone (62%)
Kalinin and Krundyshev [106]	Quantum ML methods application in high-performance intrusion detection especially for big data inputs	QSVM, QCNN, Quantum parallelism, Quantum superposition, Entanglement	Claims (98%) for accuracy in big data processing with a speed that is twice faster than that of conventional ML algorithms
Said [112]	QSVM model for detecting DDoS attacks on smart micro-grid (SMG)	QSVM, Quantum gates	High success rates and thus efficient model in terms of accuracy and computational resources consumption
Ko and Jung [113]	A new AES (advanced encryption standard) cryptographic and quantum computing encryption/decryption scheme exclusively for AES image files	Quantum gate-based AES algorithm	Overall good performance in safeguarding encryption/decryption technologies against threats
Azeez et al. [114]	QRNGs (Quantum Random Number Generators) and AI (Artificial Intelligence) integration to provide a cybersecurity boost in financial supply chains	Quantum superposition, Entanglement	Faster encryption and decryption, lower latency, and higher resistance to predictive, quantum, and brute-force attacks are among the benefits of improved cybersecurity in the financial supply chain that the combination of technologies brings

**Table 12:** Summary of research challenges and future directions.

Challenge	Description	Future Direction
Interpretability	Black-box nature of DL/RL models reduces trust in critical applications	Integrate XAI techniques (e.g., SHAP, LIME) and develop visual analytics dashboards
Computational Costs	High resource demands for training and hyperparameter optimisation (e.g., GPUs with 16 GB+ memory)	Optimise algorithms for specific hardware and explore lightweight AI for edge devices
Data Quality	Dependency on outdated or imbalanced datasets (e.g., KDDCup’99 with attack-to-normal ratios < 1:10)	Develop synthetic datasets using GANs and encourage real-time data sharing
Real-Time Application	Latency issues in resource-constrained environments (e.g., IoT, edge devices)	Advanced edge-cloud architectures and asynchronous FL protocols
Privacy Concerns	FL’s non-IID data and data privacy risks	Implement cryptographic methods (e.g., QKD) and blockchain for secure model updates
Emerging AI Techniques	Limited scalability of generative AI, neuro-symbolic AI, and swarm intelligence	Optimise integration frameworks and test on dynamic, high-dimensional datasets
Quantum computing	NISQ hardware limitations (e.g., <100 qubits, noise) and encoding overhead	Develop hybrid quantum–classical models and test on real quantum hardware
Ethical Issues	Potential biases in AI models affecting fairness	Develop fairness-aware models and ethical guidelines for cybersecurity

## Data Availability

Not applicable.
